# Exercise before, during, and after Hospitalization for Allogeneic Hematological Stem Cell Transplant: A Feasibility Randomized Controlled Trial

**DOI:** 10.3390/jcm9061854

**Published:** 2020-06-14

**Authors:** Daniel Santa Mina, Lianne B. Dolan, Jeffrey H. Lipton, Darren Au, Encarna Camacho Pérez, Alyssa Franzese, Shabbir M. H. Alibhai, Jennifer M. Jones, Eugene Chang

**Affiliations:** 1 Faculty of Kinesiology and Physical Education, University of Toronto, Toronto, ON M5S 2W6, Canada; darren.au@uhnresearch.ca (D.A.); encarna.camacho@uhnresearch.ca (E.C.P.); 2 Department of Supportive Care, Princess Margaret Cancer Centre, Toronto, ON M5G 2C1, Canada; lianne.dolan@gmail.com (L.B.D.); alyssa.franzese@uhnresearch.ca (A.F.); shabbir.alibhai@uhn.ca (S.M.H.A.); jennifer.jones@uhn.ca (J.M.J.); eugene.chang@uhn.ca (E.C.); 3 Faculty of Medicine, University of Toronto, Toronto, ON M5S 1A8, Canada; jeff.lipton@uhn.ca; 4 Toronto Rehabilitation Institute, University Health Network, Toronto, ON M5G 2A2, Canada; 5 Department of Medical Oncology and Haematology, Princess Margaret Cancer Centre, Toronto, ON M5G 2C1, Canada

**Keywords:** exercise, stem cell transplant, cancer, oncology, rehabilitation, prehabilitation

## Abstract

People with cancer who undergo allogeneic hematological stem cell transplant (allo-HSCT) experience significant deconditioning that can compromise quality of life. Exercise has shown to be beneficial before or after allo-HSCT; however, little is known about exercise therapy delivered across the continuum of care. We conducted a feasibility randomized controlled trial of exercise delivered prior to admission, during the inpatient stay, and after discharge versus control in people with planned allo-HSCT. Feasibility was assessed via recruitment and retention rates, the incidence of adverse events, and adherence to the exercise prescription. Estimates of efficacy were measured at baseline, one week prior to hospital admission, and 100 days and one year after transplant. The recruitment and retention rates were 20% and 33%, respectively. One serious adverse event occurred during the baseline six-minute walk test that precluded participation in the study and no adverse events were associated with the intervention. From baseline to pre-transplant, the intervention group improved six-minute walk test distances by 45 m (95% CI: −18.0 to 108.7)—a finding that warrants further investigation with an adequately powered trial. Our study contributes important feasibility considerations and pilot data for future exercise intervention research in allo-HSCT recipients.

## 1. Introduction

Hematological malignancies and myeloproliferative neoplasms are commonly treated with allogeneic hematopoietic stem cell transplantation (allo-HSCT). Advancement in allo-HSCT conditioning regimens, treatments for opportunistic infectious complications, and management of graft versus host disease (GVHD) have improved the five-year net survival rate for blood-related cancers [1]. Unfortunately, allo-HSCT patients experience significant reductions in physical capacity as a result of pre-transplant conditioning regimens [2,3,4,5] and post-transplant comorbidities [6] that are associated with reduced psychological wellbeing [7,8], survival [9,10], and short- and long-term health-related quality of life (HRQOL) [11,12]. Compounding this, physical capacity is further impacted by prolonged physical inactivity during hospital stays [13].

For many allo-HSCT recipients, physical capacity may take over a year to recover to pre-transplant levels and tends to remain below population norms [14,15]. Reduced physical capacity across the allo-HSCT experience is associated with greater disability, fatigue, length of hospital stay, and mortality [3,6]. Several studies have shown that exercise interventions introduced prior to or after transplant can improve physical capacity by the time of hospital discharge following HSCT, in addition to improvements in fatigue, psychosocial wellbeing, and HRQOL [16,17,18]. However, given the repeated and multifactorial physiological insult from allo-HSCT and associated treatments, employing exercise as a preventative and restorative intervention is likely an important strategy to optimize physical capacity across the treatment experience.

Exercise across the continuum of acute care for allo-HSCT recipients may best resemble an ideal clinical state but this has been sparsely examined. Given the acknowledged challenges to exercise delivery in this population [19,20,21,22], it remains important to assess the feasibility of exercise across the multiple phases of allo-HSCT. Moreover, long-term follow-up of allo-HSCT patients who exercise pre- and post-transplant are needed. Accordingly, we conducted a phase II randomized controlled trial (RCT) to assess the feasibility of an individualized exercise program delivered as prehabilitation (prior to admission), peri-transplant (during the inpatient stay), and rehabilitation (up to 100 days) for adult patients with planned allo-HSCT. Our secondary objective was to provide preliminary efficacy data on physical fitness, psychosocial wellbeing, and clinical outcomes at clinical milestone timepoints and up to one year following transplant.

## 2. Experimental Section

### 2.1. Design and Setting

This was a two-armed, feasibility RCT of exercise versus control for people with planned allo-HSCT. The trial was registered with clinicaltrials.gov (NCT02273024) and approval was obtained from the local institutional research ethics board (University Health Network, Toronto, ON, Canada). All participants provided written informed consent before engaging in any study-related activity. Facility-based exercise sessions prior to admission for transplant as well as outcome assessments were completed in the Cancer Rehab and Survivorship program at the Princess Margaret Cancer Centre. Consolidated Standards of Reporting Trials (CONSORT) 2010 checklist is provided in Supplemental Table S1.

### 2.2. Sample

Patients were recruited from the Allogeneic Stem Cell Transplant Clinic within the Cancer Centre. The inclusion criteria were: aged 17 years or older; completed induction therapy and awaiting allo-HSCT; able to ambulate independently; medically cleared to exercise by the transplant physician; willing to attend supervised exercise sessions and complete pre- and post-transplant study-related assessments; willing to be randomized; proficient in English to understand exercise testing and training instructions as well as to complete the study consent form and questionnaires. Patients were excluded if they had more than one active malignancy; had a severe or unstable neurological, cardiorespiratory, or musculoskeletal disease or mental illness that might compromise ability to perform exercise; or if there was less than one week between recruitment and scheduled admission for allo-HSCT.

### 2.3. Randomization

After providing informed consent, participants were scheduled for an appointment to complete baseline measurements and receive study arm assignment. Equal randomization (1:1) to the exercise intervention (EX) and control (CON) groups was achieved via a computer-generated random sequence list indicating group allocation. Concealed allocation was achieved as the research assistant conducting randomization received the group assignment from a co-investigator removed from the assessment and without access to participant information. Participants in both study arms received standard of care physiotherapy during the inpatient period that focused on maintaining ability to perform activities of daily living.

**Exercise Intervention (EX)**. Immediately following randomization to EX, participants received an individualized exercise prescription from an oncology-trained registered kinesiologist based on the baseline assessment. The exercise intervention was delivered in three phases: (1) prehabilitation (from up to eight weeks prior to the date of admission for transplant); (2) inpatient (before and after allo-HSCT); (3) rehabilitation (up to 100 days post-discharge). All EX participants received resistance bands to complete their resistance training during each phase of the intervention as well as an exercise diary to track adherence and monitor progression. All sessions began with a 3 to 5-min aerobic warm-up and concluded with a yoga-based stretching and a relaxation breathing routine. The training loads for each element of the exercise prescription were individually and gradually progressed or modified in an attempt to maintain sufficient training stimulus and adapt to participants’ individual needs.

During the prehabilitation phase, participants were instructed to achieve 90–150 min of exercise per week via one supervised, facility-based exercise training session with the kinesiologist and two additional unsupervised, home-based sessions. Resistance training sessions were designed to be approximately 30–45 min and included 8–10 exercises performed for 2–3 sets of 6–12 repetitions using free weights and/or resistance bands. Resistance exercises included a combination of lower body (e.g., squats, side squats, and lunges), upper body (e.g., back rows, chest fly, chest press, push up, shoulder press, lateral arm raises, biceps curls, and triceps extensions), and core exercises (e.g., “cat-cows”, Paloff press with resistance bands, modified and standard planks). Aerobic exercise was conducted for 10–15 min at 60–80% of heart rate reserve using a stationary cycle, treadmill, or elliptical machine, and brisk walking for home-based training if other aerobic training equipment was not available.

During the inpatient training phase, exercises were completed on the inpatient unit (in the patient’s room or around the unit) and occurred from the day of hospital admission to the day of hospital discharge. The inpatient exercise prescription was structurally similar to the prehabilitation phase but included lower training volumes. Resistance training was reduced to 10–30 min targeting 1–2 sets of 4–6 repetitions per exercise using the participant’s exercise bands. Aerobic training was completed on stationary cycles in the patient rooms or walking around the unit for 10–15 min per session at 60% of the heart rate reserve. Similar to the prehabilitation phase, one inpatient exercise session was supervised by the kinesiologist and two additional sessions were prescribed for participants to complete on their own.

The rehabilitation phase commenced after hospital discharge. The target training volumes and ratio of facility-based, supervised sessions to home-based, unsupervised sessions described in the prehabilitation phase were resumed. Exercise prescriptions during the rehabilitation phase were individually modified to accommodate changes in fitness or medical status. Participants were offered supervised exercise sessions once per week until 100 days post-transplant, combined with two to three at-home sessions per week. After 100 days post-transplant, participants were advised to maintain their exercise and physical activity behaviors, but no ongoing communication was provided to support or encourage participation.

**Control Group (CON)**. Participants randomized to CON received the usual care for patients within the treating facility, which included access to stationary bikes as well as exercise placards (recommending and describing basic in-room exercises) in their inpatient rooms. CON participants were also offered a home-based exercise program identical to the prehabilitation and rehabilitation phases following their 100 day post-transplant assessment. Supervised, facility-based training was not offered to CON participants.

### 2.4. Outcomes

The primary outcomes of this study pertained to determining the feasibility of conducting a phase III RCT in comparable tertiary care facilities. Feasibility was assessed via participant recruitment and retention, adverse events related to the intervention, and adherence to the prescribed exercises. Recruitment rate was calculated as the number of participants randomized divided by the number of eligible participants approached for the study. Retention rate was calculated as the proportion of retained participants in the study until the end of the trial. Adherence to EX was calculated as the proportion of the prescribed exercises that were completed during each intervention phase and separated by modality (i.e., aerobic and resistance training). Missing data for physical fitness and patient-reported outcomes were quantified as a percentage using the sample size at each time point divided by the number of completed measures at that time point.

Secondary outcomes pertained to deriving preliminary estimates of intervention effects for future sample size calculations. Study outcomes were measured at baseline (T0), approximately one week prior to admission for transplant (T1), and at 100 days (T2) and one year (T3) after transplant. Demographic information and medical characteristics were collected at baseline by questionnaire and chart review, respectively. Medical characteristics included diagnosis; time from diagnosis to transplant date; the pre-transplant conditioning regimen; severity of symptoms using the Memorial Symptom Assessment Scale (MSAS) [23]; length of hospital stay from the date of transplant to hospital discharge; incidence of acute and chronic GVHD, and the use of steroids for GVHD symptom management (yes or no).

Peak aerobic capacity (VO2 peak) was measured using a cardiopulmonary exercise test (CPET) to volitional fatigue [24]. Breath-by-breath gas exchange was measured using the TrueOne 2400 gas analyzer (ParvoMedics, Salt Lake City, UT, USA) and was conducted according to standardized, joint American Thoracic Society American/American College of Chest Physicians testing protocols with the exception of physician presence or electrocardiography [25]. Functional aerobic capacity was assessed using the six-minute walk test (6 MWT) [26] and the 30-s chair-stand test [27]. Body mass index was calculated by dividing weight (kg) by the squared product of height (m) and body fat percentage was estimated using bioelectric impedance analysis (TBF-300 A, Tanita, Tokyo, Japan). Grip strength was measured using a hand dynamometer (Sammons Preston, Bolingbrook, IL, USA) according to established protocols [28]. Upper extremity strength was measured using maximal voluntary isometric contraction (MVIC) of the elbow flexors and extensors using a digital handheld dynamometer (MicroFET2, Hoggan Scientific, Salt Lake City, UT, USA). The strength testing protocol required participants to be seated with their elbow positioned at 90 degrees with their hand either positioned on the underside (flexion) or topside (extension) of an anchored table. The dynamometer was placed on the palm of the participant, who was instructed to gradually generate force against the dynamometer for 2 s and maintain a maximal effort for an additional 5 s. Two trials were completed for each arm, with the maximum value per arm recorded to the nearest kilogram.

HRQOL was captured via the European Organization for the Research and Treatment of Cancer Quality of Life Questionnaire (EORTC QLQ-C30) [29,30]. Anxiety was assessed via the Generalized Anxiety Disorder (GAD7) [31] and severity of depression was measured using the Patient Health Questionnaire (PHQ-9) [32]. Fatigue was assessed via the Functional Assessment of Cancer Therapy-Fatigue (FACT-F) [33,34] and the Multidimensional Fatigue Inventory (MFI) [35]. To measure self-efficacy to perform exercise in spite of difficult circumstances, the spinal cord injury Exercise Self-Efficacy Scale (ESES) [36] was employed, given the otherwise generic nature of the questions (e.g., “I am confident that I can overcome barriers and challenges with regard to physical activity and exercise if I try hard enough”).

### 2.5. Statistical Analysis

Feasibility outcomes, participant demographics and disease characteristics were summarized using descriptive statistics (mean ± standard deviation, SD; or frequency and percentages). Chi-square (or Fisher’s exact test, where appropriate) and independent samples t-tests were used to compare baseline demographic and disease characteristic data between EX and CON. Changes in outcomes over time were assessed using within-group and between-group comparisons via linear mixed-effect models, and the means at given timepoints and the deltas between timepoints are reported with their respective 95% confidence intervals (95% CI). Maximum likelihood estimations were used to estimate the adjusted sample mean scores of all physical functioning and patient-reported outcomes, except the chair-stand test, length of hospitalization in days (from transplant to discharge) and days to engraftment, which were modelled using a linear mixed-effect under the Poisson distribution test for count data. All models included a group × timepoint as the fixed effect and individual participants as the random effect. Pairwise comparisons between time points (within-groups) were adjusted with Tukey’s HSD. Direct comparison of the outcomes between was not performed due to sample size constraints and risk of type I error. All analyses were done in R version 3.6.1 (R Foundation for Statistical Computing, Vienna, Austria).

## 3. Results

**Figure 1:**
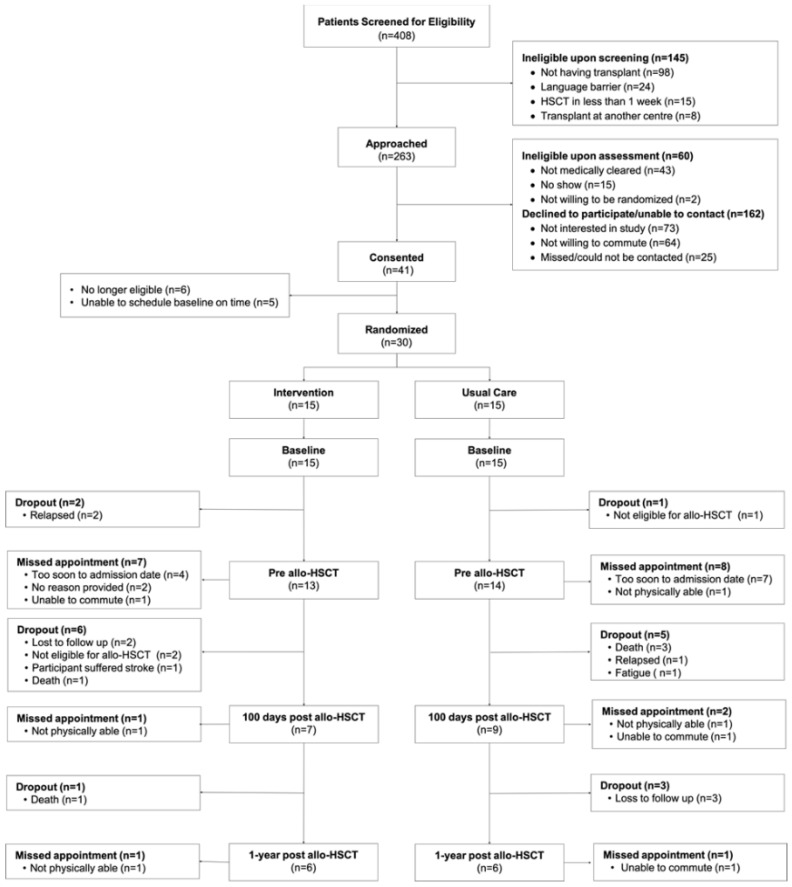
Consolidated Standards of Reporting Trials (CONSORT) Diagram

Recruitment occurred over 41 months between October 2014 and October 2018, which included an approximate seven-month recruitment hiatus due to study staff changes between July 2015 and March 2016. Reasons for ineligibility, declined participation, attrition, and missed assessments at each time point are described in the CONSORT diagram (Figure 1). Briefly, 408 patients were screened for eligibility, of whom, 263 potentially eligible patients were approached, yielding 203 patients with confirmed eligibility. Of these 203 participants, 41 patients provided consent to participate in the study (20.2%); however, only 30 proceeded to be randomized given changes in eligibility status or transplant scheduling. From baseline to the 100 days post-transplant, 47% and 60% were retained in the study in the EX and CON groups, respectively. By one year post-transplant, 33% in each group were retained in the study.

Baseline demographic, disease, and transplant characteristics were similar between groups and are presented in Table 1. Briefly, the mean age of the participants was 50.4 ± 18.1 years and 48.4 ± 13.0 years for the EX and CON groups, respectively (*p* = 0.730). Two-thirds of the participants in both groups were white, and a majority of participants reported being in a married or common law relationship, had an undergraduate degree, and had a household income of greater than $80,000 CAD. Most participants had a planned allo-HSCT for leukemia (9 and 13 in the EX and CON, respectively). There was one non-cancer case (mitochondrial neurogastrointestinal encephalopathy) with an indication for allo-HSCT in the CON group. Of those that received transplantation, the majority underwent fludarabine, busulfan, and total body irradiation of 200 cGy (FBT200) reduced-intensity conditioning. GVHD prophylactic treatment occurred for all patients and most commonly comprised anti-thymocyte globulin, post-transplant cyclophosphamide, cyclosporine (ATG-PTCy-CSA). The length of hospital stay from transplant to discharge was 27.4 ± 3.8 days and 28.6 ± 3.5 days for the EX and CON group, respectively (*p* = 0.813). Days to engraftment was 16.8 ± 1.5 and 17.6 ± 1.1 for the EX and CON group, respectively (*p* = 0.628).

The median duration of each phase of the trial for the overall cohort and by group is shown in Table 2. For the EX group, median exercise intervention length was 36 days during the prehabilitation phase; 30 days for the inpatient phase; 95 days from post-discharge to the T3 time point (medians are presented as the data were not distributed normally; duration of study phases were not statistically different between study arms). There was one serious adverse event associated with study participation which included a fall during the baseline 6 MWT resulting in a subarachnoid hemorrhage and admission to the hospital for surveillance for two days. The participant was discharged and was not randomized.

EX group adherence to aerobic and resistance exercise prescriptions across each intervention phase, as well as total number of aerobic minutes, are shown in Table 3. Exercise logbooks were not consistently completed or submitted by participants at each stage of the intervention. For those that did submit their exercise logbooks, participants reported completing a median of approximately 55% of their aerobic exercise prescription and 99% of their resistance training prescription per week during the prehabilitation and inpatient phases of the intervention relative to the participants’ exercise prescription. Following discharge and up to 100 days after transplant, the median weekly adherence to aerobic and resistance training prescriptions was 20 and 100%, respectively. During the prehabilitation and inpatient phases, the median weekly number of minutes spent doing aerobic activity was 50, whereas, during the rehabilitation phase, it reduced to 18 min per week.

We report the volume of missing data for the outcome measures for all retained participants at each time point in Supplemental Table S2. During follow-up visits, missing data ranged from 14 to 62% across all measures. Missing data varied across time points for both groups, where a majority were due to missed appointments. Those that were able to attend the scheduled study visits were able to complete the majority of the outcome measures.

**Figure 2:**
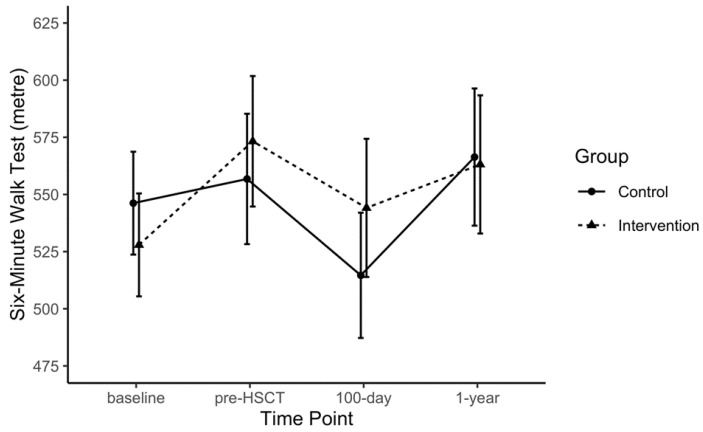
Six-minute Walk Test distance over time for the intervention and control groups.

**Figure 3:**
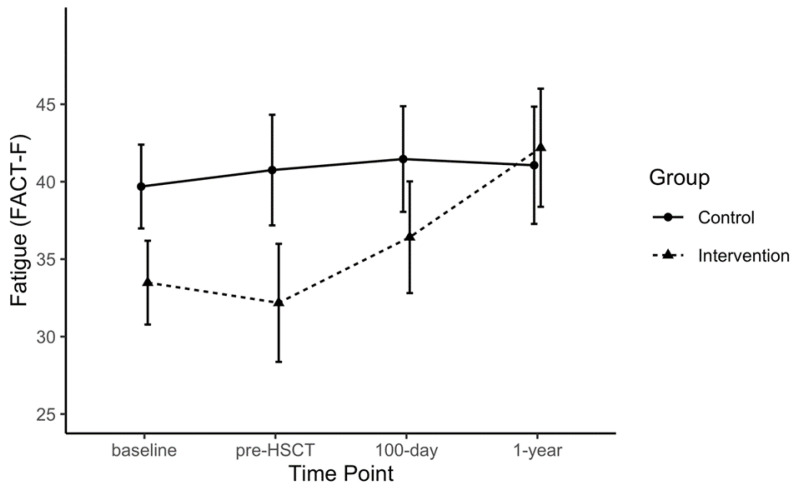
Fatigue score measured by Functional Assessment of Cancer Therapy: Fatigue (FACT-F) over each time point by group.

**Figure 4:**
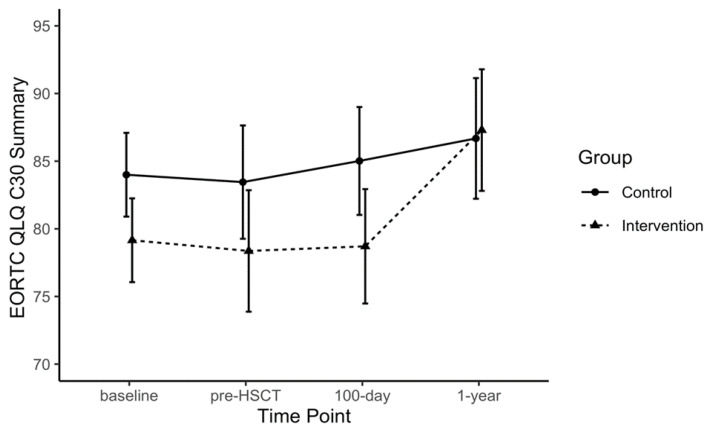
Quality of life summary score measured by European Organization for the Research and Treatment of Cancer Quality of Life Questionnaire (EORTC QLQ-C30) over each time point by group.

Point estimates per time point, as well as within- and between-group differences for each outcome, are provided in the Supplemental Materials (Supplemental Tables S3–S6) for the purposes of future trial design. Some data signals related to exploratory comparative analyses are highlighted here; however, due the small sample size and to differences in baseline values across several outcomes, only notable within-group observations are described. The 6 MWT distances increased from baseline to pre-HSCT in the EX group beyond reported minimal clinically important differences (MCID) [37,38] and both groups displayed a reduction in 6 MWT distances from pre-HSCT to 100 days post-HSCT (EX: −29.1 m, 95% CI: −110.5 to 52.2; CON: 42.2 m, 95%CI: −113.2 to 28.9; Figure 2). Large magnitude within-group changes were also observed for the FACT-F in the EX group from pre-HSCT to one year post-HSCT (FACT-F: +10.0, 95% CI: −1.4 to 21.4; MFI: +6.1, 95% CI: −2.1 to 14.3; Figure 3). Handgrip strength showed a significant reduction from baseline to 100 days post-HSCT in CON (−13.4 kg, 95% CI: −22.4 to −4.4). The MVIC of the biceps declined in the CON group from baseline and pre-HSCT levels to the 100 days post-transplant measurement by ~6 kg. At one year post-transplant, QLQ-C30 summary scores were improved by greater than 8 points compared to baseline, pre-HSCT, and 100 days post-transplant levels (Figure 4), where an MCID of 7.6 points has been suggested in multiple myeloma patients [39]. The EX group reported improved scores on the reduced-motivation scale of the MFI by approximately 3 points at 100 days post-transplant compared to baseline and pre-transplant levels. Due to many patients not obtaining VO2 peak values that represented maximal aerobic capacity, VO2 at anaerobic threshold per group remained relatively stable for both groups from baseline to 100 days post-transplant and increased one year after transplant.

## 4. Discussion

We examined the feasibility of delivering exercise before and after allo-HSCT, including during the inpatient stay. Our findings and experiences raise important considerations for conducting an RCT of a similar nature within a comparable urban, academic healthcare institution. We recruited only 20% of eligible participants for our trial, primarily owing to a disinterest in participating in the study or unwillingness to commute for the study-related training and/or testing. Since our study was initiated, the evidence regarding the safety and benefits of exercise for people undergoing allo-HSCT has grown considerably. Compared to our study, where 70% of otherwise eligible participants did not receive medical clearance to participate, the evolved evidence and awareness of exercise tolerability and benefits for allo-HSCT patients may result in greater participation in future studies. Our recruitment rate starkly contrasts with the only previous comparable RCT by Wiskemann and colleagues [40], which reported a recruitment rate of nearly 80% of eligible participants. Nevertheless, our experience is consistent with a recent feasibility study of exercise delivered prior to allo-HSCT [22] as well as the broader oncology clinical research that finds participation trials to be unachievable for approximately three quarters of the target population due to structural and clinical barriers [41]. While our findings partially reiterate previous reports that emphasize strategies to minimize facility visits, the financial costs, or inconvenience of facility-based training [42,43], more research is needed to better understand environmental, socioeconomical, and psychosocial barriers to exercise for the allo-HSCT population.

Attrition in our study was nearly 70%, commonly attributed to factors that relate to the clinical complexity of the population (e.g., relapse, death, comorbidities or symptom burden). These highlight the challenge of conducting longitudinal research in this population and the value of using mortality and relapse as outcome measures for those whose diagnosis brings precarious short-term survival rates. Facilitating ease of data collection and intervention participation may reduce attrition rates for reasons that relate to inability to return the facility, which may also improve data completeness, given the number of visits due to being physically unable to attend.

Despite recruitment and retention issues, the intervention appeared to engage those that did participate, demonstrated by adherence to the exercise prescription, particularly for resistance training. Resistance training may represent a more feasible option for allo-HSCT patients, given that exercises can be adapted to be done in bed or in a chair in the patient’s room with minimal equipment (e.g., resistance bands). Moreover, with light resistance bands, the exercise prescription can be maintained in many circumstances despite significant deconditioning or symptom burden. Alternatively, for aerobic exercise, the minimum physiological load for even gentle exercises (slow-paced cycling or walking) may exceed their perceived tolerability. Although participants in this study had stationary cycles in their inpatient rooms, the ability to perform aerobic exercises in other settings may not have this provision, or may have restricted ability to walk outside or with others due to compromised immune function or while in isolation.

At baseline, participants in this study reported poorer FACT-F [44,45] and 6 MWT [46,47] scores when compared with healthy individuals, highlighting the need for improved physical capacity in this sample. While our study was not designed to determine efficacy, we note clinically salient within-group improvements in FACT-F and 6 MWT scores, as well as grip strength and quality of life, across various timepoints for EX participants. These support the need for trials scaled for efficacy on these important outcomes that have prognostic clinical value [3,6]. We provide a comprehensive set of point estimates, within- and between-group contrasts, and their respective measures of variability to advise future sample size calculations. We also note that there were no adverse events associated with the intervention. These underscore previous findings of safety and tolerability of exercise for allo-HSCT recipients. However, a serious adverse event associated with 6 MWT in one-participant prior to randomization is important to acknowledge. Acute, traumatic adverse events of such severity during the 6 MWT are rarely reported; while all safety protocols and responses were adhered in the present protocol, the event reinforces the need for clear instructions, constant monitoring for potential fall risk or injury, and environmental suitability for this test that is widely accepted as safe and low-risk.

Only Wiskemann and colleagues [40] have similarly examined structured exercise programming pre-, during, and post-allo-HSCT, and did so via a multicenter RCT in 105 participants. Intervention participants received an unsupervised, home-based exercise program prior to hospital admission (1–4 weeks), supervised exercise programming during the inpatient period, and 6–8 weeks of unsupervised, home-based exercise following hospital discharge. The control group received pedometers, were advised on the benefits of moderate physical activity, and were offered physiotherapy three times per week during hospitalization. At 6–8 weeks post-discharge, intervention participants demonstrated less general and physical fatigue (measured using the MFI−20), and greater self-reported physical function (EORTC-QLQ-C30 subscale) and 6 MWT scores compared to control participants. Their trial demonstrated feasibility of a multi-setting and multiphasic exercise intervention, with >80% compliance to the exercise prescription, nearly 80% retention to the final study assessment, and minimal missing data. Our data reflect poorer metrics of feasibility which may relate to institutional or population differences in patients’ and healthcare providers’ readiness for exercise interventions in allo-HSCT recipients at the time of our study. Our between-group comparisons are hampered due to sample size limitations and compounded by group-level differences at baseline; nevertheless, our within-group findings of improvement in fatigue, HRQOL, and 6 MWT in the intervention group (and lack thereof in the control group) are generally consistent with the findings by Wiskemann et al. and warrant further examination [40].

It is worth reviewing potential lessons learned from our experiences to support the design of future studies. First, recruitment strategies must account for the variability in patient conditioning regimens and health trajectory during the pre-transplant period. Prehabilitation may be feasible for some; however, transplant status may change following recruitment, where the prehabilitation goals or duration must be altered or no longer apply. Second, our sample was prone to discontinuing the CPET prior to achieving values that approximate maximum aerobic capacity. Challenges of conducting CPETs in people with cancer and the potential implications of underestimating aerobic capacity have been recently described [48,49]. In the current study, we report anaerobic threshold, complemented by the 6 MWT and 30 s chair sit-to-stand test to describe functional fitness, which may be more practical in some settings. Third, scheduling for visits for facility-based assessments was often difficult due to the frequency of medical appointments and general physical wellness of the participant. Home-based assessments that can be performed reliably may facilitate data collection and minimize participant burden. Fourth, exercise in the inpatient setting and up to 100 days post-transplant required significant adaptation to the exercise prescription to accommodate deconditioning, such as modifying resistance exercises so they could be performed in bed while on the unit. This was critically supported through discussions with unit clinical staff to advise in-room exercise prescriptions and training sessions.

Interpretation of our findings is cautioned due to the following: (i) small sample size with significant attrition to the final data collection timepoint; (ii) conducted in a single center; (iii) non-blinded participants and outcome assessors; (iv) poor completion rate of the exercise logbooks; (v) challenges to achieving accurate estimates of maximal aerobic capacity. Moreover, while physiotherapy was available as the standard of care for all participants to assist with maintenance of activities of daily living and general conditioning, the volume and nature of individual participants’ physiotherapy treatments was not captured, potentially confounding the results. Subsequent trials would be enhanced through the use of routine check-ins with participants from 100 days to one year post-transplant, the use of remote data capture for self-report items to optimize data collection for participants, and employing digital tools to capture intervention adherence (e.g., accelerometry or electronic apps for exercise logging). Furthermore, the optimal timing, setting, and modality for exercise interventions with allo-HSCT recipients remains an important question with several different factors potentially influencing the feasibility and efficacy of exercise in the pre-treatment, inpatient, and post-transplant outpatient periods.

## 5. Conclusions

This RCT examined the feasibility of exercise across the acute allo-HSCT experience and up to one year post transplant while capturing pilot data related to its effects on physical and psychosocial performance. Our feasibility data highlight the challenging nature of exercise interventions in this setting, emphasizing considerations for inclusion criteria, intervention setting and timing, and outcome selection—all of which can influence trial duration and cost. Nevertheless, our study findings support the tolerability and safety of exercise for allo-HSCT recipients across various stages of their treatment timeline and note potential pragmatic advantages for resistance training in this population. Further research is required to confirm the potential benefits of exercise versus control on fatigue and physical function observed in intervention in allo-HSCT recipients.

## Figures and Tables

**Table 1:** Baseline demographic, disease, and treatment characteristics for intervention and control.

Characteristic	Intervention (*N* = 15)	Control (*N* = 15)	*p*-Value
Age (mean [SD])	50.4 (18.1)	48.4 (13.0)	0.730
Gender (male [%])	8 (53.3)	7 (46.7)	1.00
BMI (mean [SD])	24.6 (3.3)	26.1 (5.1)	0.345
Disease (%)			0.145
Leukemia	9 (60.0)	13 (86.7)	
Lymphoma	1 (6.7)	0 (0.0)	
Myelodysplastic syndrome	5 (33.3)	1 (6.7)	
Mitochondrial neurogastrointestinal encephalopathy syndrome	0 (0.0)	1 (6.7)	
Ethnicity (%)			0.333
East Asian	2 (13.3)	0 (0.0)	
West Asian	2 (13.3)	0 (0.0)	
South Asian	0 (0.0)	1 (6.7)	
White/Caucasian	10 (66.7)	10 (66.7)	
Black	0 (0.0)	1 (6.7)	
Jewish	1 (6.7)	1 (6.7)	
South East Asian	0 (0.0)	1 (6.7)	
West Indian	0 (0.0)	1 (6.7)	
Income (%)			0.842
<40,000	3 (21.4)	2 (14.3)	
40,000–80,000	3 (21.4)	4 (28.6)	
>80,000	8 (57.1)	8 (57.1)	
Marital status (%)			0.909
Single/never married	4 (26.7)	3 (20.0)	
Married/common law	10 (66.7)	11 (73.3)	
Divorced	1 (6.7)	1 (6.7)	
Education (%)			0.74
High school graduate	1 (6.7)	3 (20.0)	
Community college/trade school graduate	1 (6.7)	1 (6.7)	
University graduate	10 (66.7)	9 (60.0)	
Graduate university degree	3 (20.0)	2 (13.3)	
Work status (%)			0.058
Retired	4 (26.7)	0 (0.0)	
Unemployed	2 (13.3)	0 (0.0)	
Part-time	1 (6.7)	2 (13.3)	
Full-time	7 (46.7)	13 (86.7)	
Student	1 (6.7)	0 (0.0)	
Donor type ^†^			0.179
Human leukocyte antigen matching related	5 (45.5)	8 (57.1)	
Human leukocyte antigen matching unrelated	5 (45.5)	3 (21.4)	
Haploidentical	0 (0.0)	3 (21.4)	
Missing	1 (9.1)	0 (0.0)	
HSCT conditioning protocol (%)			0.18
ECOG-2993 plus DASATINIB	1 (6.7)	0 (0.0)	
FBT(200)	9 (60.0)	14 (93.3)	
FBT(400)	1 (6.7)	0 (0.0)	
no HSCT *	4 (26.7)	1 (6.7)	
GVHD prophylaxis protocol (%)			0.172
ATG-CSA-MMF	2 (13.3)	0 (0.0)	
ATG-PTCY-CSA	8 (53.3)	13 (86.7)	
CSA-MTX	1 (6.7)	1 (6.7)	
no HSCT *	4 (26.7)	1 (6.7)	
Presence of acute GVHD (%)	5 (33.3)	8 (53.3)	0.461
Presence of chronic GVHD (%)	3 (20.0)	2 (13.3)	1.0
Use of steroids for GVHD symptom management (%)	5 (33.3)	3 (20.0)	0.68

ATG: anti-thymocyte globulin; BMI: body mass index; CSA: cyclosporine A; MMF: mycophenolate mofetil; ECOG: Eastern Cooperative Oncology Group; FBT: fludarabine, busulfan, total body irradiation; GVHD: graft versus host disease; HSCT: hematopoietic stem cell transplant; MTX: methotrexate; PTCY: post transplant cyclophosphamide. * withdrawn from study due to change in eligibility status; ^†^ Excludes participants not eligible for transplant.

**Table 2:** Median duration in days for each phase of the trial.

	Group	Median Days (IQR)	*p*-Value ^†^
Prehabilitation	Intervention (*n* = 11)	36.0 (55)	0.728
	Control (*n* = 13)	34.0 (54)	
	Overall (*n* = 24)	35.0 (58.25)	
Inpatient	Intervention (*n* = 11)	30.0 (10.5)	0.816
	Control (*n* = 12)	27.0 (25.0)	
	Overall (*n* = 23)	28 (19.3)	
Rehabilitation	Intervention (*n* = 6)	95.0 (31.5)	0.389
	Control (*n* = 7)	74.0 (19.5)	
	Overall (*n* = 13)	83 (36)	

Prehabilitation refers to duration of time from the baseline assessment to admittance date prior to transplant; Inpatient refers to the duration of time admitted to the hospital for the transplant; Rehabilitation refers to the duration of time from discharge to 100 days post-HSCT measured as days from transplant to T3 follow-up date; ^†^ Unpaired Mann–Whitney test.

**Table 3:** Median adherence to aerobic and strength training exercise prescription and total aerobic minutes by intervention phase.

Weekly Exercise Adherence		
Phase	Exercise Prescription Component	Median (IQR)
Prehabilitation (*n* = 9)	Aerobic (%)	56 (7 to 100)
	Resistance (%)	99 (52 to 100)
	Total aerobic minutes per week	50 (6 to 147)
Inpatient (*n* = 7)	Aerobic (%)	55 (21.9 to 97)
	Resistance (%)	99 (54 to 100)
	Total aerobic minutes	50 (19.75 to 95)
Rehabilitation (*n* = 3)	Aerobic (%)	20 (0 to 53)
	Resistance (%)	100 (97 to 100)
	Total aerobic minutes	18 (0 to 48)

% is calculated as the proportion of the participant’s weekly exercise prescription that was completed (all intervention weeks per phase were averaged to provide a single adherence value). Participants were prescribed a minimum of 90 min per week of aerobic activity.

## Supplementary Materials

The following are available online at https://www.mdpi.com/2077-0383/9/6/1854/s1, Table S1: CONSORT 2010 checklist of information to include when reporting a randomised trial *.Table S2: Frequency and percentage of missing outcomes for all retained participants, Table S3: Mean estimates ± SE and between-group difference for physical measures at each time point, Table S4: Within-group differences across time points for physical fitness measures, Table S5: Mean estimates ± SE and between-group differences for patient-reported outcomes at each time point, Table S6: Within-group differences across time points for patient-reported outcomes.
