# Supplementary material for: Exercise before, during, and after Hospitalization for Allogeneic Hematological Stem Cell Transplant: A Feasibility Randomized Controlled Trial
Source: J Clin Med. 2020 Jun 14;9(6):1854. doi: 10.3390/jcm9061854 (PMC7355733; doi:10.3390/jcm9061854)
Supplement: Supplementary file 1 [file jcm-09-01854-s001.zip › HSCT_supplementary_tables_June13_2020.docx]

| Table S2: Frequency and percentage of missing outcomes for all retained participants. | | | | | | | | |
| --- | --- | --- | --- | --- | --- | --- | --- | --- |
| **Outcome** | **Intervention** | | | | **Control** | | | |
|  | **Baseline**  **(n=15)** | **Pre-HSCT**  **(n=13)** | **100 day**  **(n=7)** | **1 year**  **(n=6)** | **Baseline**  **(n=15)** | **Pre-HSCT**  **(n=14)** | **100 day**  **(n=9)** | **1 year**  **(n=6)** |
| Six-Minute Walk Test | 0 (0) | 7 (54) | 2 (29) | 1 (17) | 0 (0) | 8 (57) | 2 (22) | 1 (17) |
| CPET | 2 (13) | 8 (62) | 2 (29) | 1 (17) | 5 (33) | 10 (71) | 4 (44) | 3 (5) |
| Grip strength | 0 (0) | 7 (54) | 1 (14) | 1 (17) | 0 (0) | 8 (57) | 2 (22) | 1 (17) |
| Sit-to-stand | 1 (7) | 7 (54) | 1 (14) | 1 (17) | 0 (0) | 8 (57) | 2 (22) | 1 (17) |
| Body fat | 1 (7) | 8 (62) | 1 (14) | 1 (17) | 0 (0) | 8 (57) | 2 (22) | 1 (17) |
| Waist circumference | 1 (7) | 7 (54) | 1 (14) | 1 (17) | 0 (0) | 8 (57) | 2 (22) | 1 (17) |
| FACT-F | 0 (0) | 8 (62) | 1 (14) | 1 (17) | 0 (0) | 8 (57) | 2 (22) | 1 (17) |
| MSAS Global Distress Index | 0 (0) | 7 (54) | 1 (14) | 1 (17) | 0 (0) | 8 (57) | 2 (22) | 1 (17) |
| MSAS Physical | 0 (0) | 7 (54) | 1 (14) | 1 (17) | 0 (0) | 8 (57) | 2 (22) | 1 (17) |
| MSAS Psychological | 0 (0) | 8 (62) | 1 (14) | 1 (17) | 0 (0) | 8 (57) | 2 (22) | 1 (17) |
| QLQ-C30 | 0 (0) | 8 (62) | 1 (14) | 1 (17) | 0 (0) | 8 (57) | 2 (22) | 1 (17) |
| ESES | 1 (7) | 7 (54) | 1 (14) | 1 (17) | 0 (0) | 8 (57) | 2 (22) | 1 (17) |
| GAD7 | 0 (0) | 7 (54) | 1 (14) | 1 (17) | 1 (7) | 8 (57) | 2 (22) | 1 (17) |
| PHQ | 1 (7) | 7 (54) | 2 (29) | 1 (17) | 0 (0) | 8 (57) | 2 (22) | 1 (17) |
| MFI | 0 (0) | 7 (54) | 1 (14) | 1 (17) | 0 (0) | 8 (57) | 2 (22) | 2 (33) |
| CPET: cardiopulmonary exercise test; ESES: Exercise Self-Efficacy Scale; FACT-F: Functional assessment of Cancer Therapy-Fatigue; GAD7: Generalized Anxiety Disorder; MFI: Multidimentioanl Fatigue Inventory; MSAS: Memorial Symptom Assessment Scale; PHQ: Patient Health Questionnaire; QLQ-C30: European Organization for the Research and Treatmnet of Cancer Quality of Life Questionnaire. | | | | | | | | |

| Table S3: Mean estimates ± SE and between-group difference for physical measures at each time point. | | | | | |
| --- | --- | --- | --- | --- | --- |
| **Outcome** | **Time point** | **Group** | | **Between-group contrasts**  **(Intervention – Control)** | |
|  |  | **Intervention (n=15)**  Mean estimates ± SE | **Control (n=15)**  Mean estimates ± SE | **Δ** | **(95% CI)** |
| Six-Minute Walk Test  (metres) | Baseline | 527.9 ± 22.5 | 546.2 ± 22.5 | -18.3 | (-82.6, 46.1) |
|  | Pre-HSCT | 573.3 ± 28.5 | 556.8 ± 28.5 | 16.5 | (-64.1, 97.0) |
|  | 100-day post-HSCT | 544.1 ± 30.2 | 514.6 ± 27.4 | 29.5 | (-52.0, 110.9) |
|  | 1-year post-HSCT | 563.1 ± 30.2 | 566.4 ± 30.0 | -3.2 | (-88.2, 81.7) |
| VO_2_ at Anaerobic Threshold  (ml/kg/min) | Baseline | 13.2 ± 0.9 | 14.4 ± 1.1 | -1.2 | (-4.0, 1.7) |
|  | Pre-HSCT | 13.2 ± 1.0 | 15.4 ± 1.1 | -2.2 | (-5.3, 0.8) |
|  | 100-day post-HSCT | 13.0 ± 1.1 | 14.3 ± 1.1 | -1.3 | (-4.4, 1.8) |
|  | 1-year post-HSCT | 14.1 ± 1.1 | 16.1 ± 1.2 | -2.0 | (-5.2, 1.2) |
| Chair-stand test*  (repetitions) | Baseline | 15.3 ± 1.5 | 14.8 ± 1.4 | 1.0 | (0.8, 1.3) |
|  | Pre-HSCT | 17.3 ± 2.4 | 16.8 ± 2.2 | 1.0 | (0.7, 1.5) |
|  | 100-day post-HSCT | 18.4 ± 2.4 | 14.4 ± 1.8 | 1.3 | (0.9, 1.8) |
|  | 1-year post-HSCT | 19.6 ± 2.6 | 17.5 ± 2.4 | 1.1 | (0.8, 1.6) |
| Grip strength  (kg-force) | Baseline | 64.9 ± 6.7 | 67.2 ± 6.7 | -2.3 | (-21.6, 16.9) |
|  | Pre-HSCT | 69.1 ± 7.2 | 66.6 ± 7.2 | 2.5 | (-18.1, 23.1) |
|  | 100-day post-HSCT | 64.3 ± 7.2 | 53.8 ± 7.1 | 10.5 | (-10.0, 30.9) |
|  | 1-year post-HSCT | 75.0 ± 7.4 | 70.6 ± 7.3 | 4.4 | (-16.5, 25.4) |
| Bicep maximal voluntary contraction  (kg-force) | Baseline | 23.8 ± 3.0 | 24.9 ± 2.9 | -1.1 | (-9.5, 7.3) |
|  | Pre-HSCT | 23.9 ± 3.7 | 25.3 ± 3.7 | -1.4 | (-11.9, 9.0) |
|  | 100-day post-HSCT | 27.8 ± 3.7 | 19.1 ± 3.7 | 8.7 | (-1.8, 19.2) |
|  | 1-year post-HSCT | 26.2 ± 3.9 | 25.8 ± 3.9 | 0.4 | (-10.6, 11.5) |
| Triceps maximal voluntary contraction (kg-force) | Baseline | 23.1 ± 2.8 | 22.4 ± 2.5 | 0.6 | (-7.0, 8.3) |
|  | Pre-HSCT | 24.1 ± 3.3 | 24.1 ± 2.9 | 0.0 | (-8.7, 8.8) |
|  | 100-day post-HSCT | 19.9 ± 3.3 | 17.6 ± 2.9 | 2.3 | (-6.5, 11.2) |
|  | 1-year post-HSCT | 21.8 ± 3.2 | 23.5 ± 3.0 | -1.7 | (-10.5, 7.0) |
| Body fat  (%) | Baseline | 24.9 ± 2.5 | 26.7 ± 2.4 | -1.7 | (-8.8, 5.4) |
|  | Pre-HSCT | 26.3 ± 2.8 | 27.0 ± 2.7 | -0.8 | (-8.5, 7.0) |
|  | 100-day post-HSCT | 21.7 ± 2.7 | 21.0 ± 2.6 | 0.7 | (-6.9, 8.4) |
|  | 1-year post-HSCT | 25.0 ± 2.8 | 26.4 ± 2.7 | -1.4 | (-9.3, 6.5) |
| Waist circumference  (cm) | Baseline | 91.1 ± 3.0 | 90.9 ± 3.0 | 0.3 | (-8.3, 8.9) |
|  | Pre-HSCT | 90.5 ± 3.4 | 91.3 ± 3.4 | -0.7 | (-10.3, 8.9) |
|  | 100-day post-HSCT | 85.1 ± 3.4 | 86.0 ± 3.3 | -0.9 | (-10.4, 8.6) |
|  | 1-year post-HSCT | 86.9 ± 3.5 | 90.1 ± 3.5 | -3.2 | (-13.0, 6.7) |
| *estimates for sit-to-stand are presented in counts and between-group comparisons are presented as a rate ratio. | | | | | |

| Table S4: Within-group differences across time points for physical fitness measures. | | | | | |
| --- | --- | --- | --- | --- | --- |
| **Outcome** | **Contrast** | **Group** | | | |
|  |  | **Intervention (n=15)** | | **Control (n=15)** | |
|  |  | **Estimate** | **95% CI** | **Estimate** | **95% CI** |
| Six-Minute walk test  (metres) | Baseline to Pre-HSCT | 45.3 | (-18.0, 108.7) | 10.6 | (-52.7, 73.8) |
|  | Baseline to 100-day post-HSCT | 16.2 | (-52.5, 84.8) | -31.6 | (-91.2, 28.1) |
|  | Pre-HSCT to 100-day post-HSCT | -29.1 | (-110.5, 52.2) | -42.2 | (-113.2, 28.9) |
|  | Baseline to 1-year post-HSCT | 35.2 | (-33.5, 103.8) | 20.2 | (-47.8, 88.1) |
|  | Pre-HSCT to 1-year post-HSCT | -10.1 | (-91.5, 71.2) | 9.6 | (-65.5, 84.7) |
|  | 100-day post-HSCT to 1-year post-HSCT | 19 | (-57.5, 95.5) | 51.7 | (-19.3, 122.8) |
| VO_2_ at anaerobic threshold  (ml/kg/min) | Baseline to Pre-HSCT | -0.1 | (-1.7, 1.5) | 1.0 | (-0.7, 2.7) |
|  | Baseline to 100-day post-HSCT | -0.3 | (-2.0, 1.5) | -0.1 | (-1.7, 1.5) |
|  | Pre-HSCT to 100-day post-HSCT | -0.2 | (-2.3, 1.9) | -1.1 | (-3.0, 0.8) |
|  | Baseline to 1-year post-HSCT | 0.9 | (-0.9, 2.6) | 1.6 | (-0.3, 3.6) |
|  | Pre-HSCT to 1-year post-HSCT | 0.9 | (-1.2, 3.1) | 0.7 | (-1.5, 2.8) |
|  | 100-day post-HSCT to 1-year post-HSCT | 1.1 | (-0.8, 3.0) | 1.8 | (-0.2, 3.8) |
| Chair-stand test* | Baseline to Pre-HSCT | 1.1 | (0.8, 1.6) | 1.1 | (0.8, 1.6) |
|  | Baseline to 100-day post-HSCT | 1.2 | (0.9, 1.7) | 1.0 | (0.7, 1.3) |
|  | Pre-HSCT to 100-day post-HSCT | 1.1 | (0.7, 1.6) | 0.9 | (0.6, 1.3) |
|  | Baseline to 1-year post-HSCT | 1.3 | (0.9, 1.8) | 1.2 | (0.8, 1.7) |
|  | Pre-HSCT to 1-year post-HSCT | 1.1 | (0.7, 1.7) | 1.0 | (0.7, 1.5) |
|  | 100-day post-HSCT to 1-year post-HSCT | 1.1 | (0.8, 1.5) | 1.2 | (0.8, 1.8) |
| Grip strength  (kg-force) | Baseline to Pre-HSCT | 4.2 | (-5.3, 13.8) | -0.6 | (-10.1, 8.9) |
|  | Baseline to 100-day post-HSCT | -0.6 | (-10.2, 9.0) | -13.4 | (-22.4, -4.4) |
|  | Pre-HSCT to 100-day post-HSCT | -4.9 | (-16.2, 6.5) | -12.8 | (-23.4, -2.2) |
|  | Baseline to 1-year post-HSCT | 10.2 | (-0.2, 20.5) | 3.4 | (-6.8, 13.7) |
|  | Pre-HSCT to 1-year post-HSCT | 5.9 | (-6.3, 18.1) | 4.0 | (-7.2, 15.2) |
|  | 100-day post-HSCT to 1-year post-HSCT | 10.8 | (0.1, 21.5) | 16.8 | (6.3, 27.4) |
| Bicep maximal voluntary contraction  (kg-force) | Baseline to Pre-HSCT | 0.1 | (-8.1, 8.3) | 0.4 | (-7.9, 8.6) |
|  | Baseline to 100-day post-HSCT | 4.0 | (-4.3, 12.2) | -5.8 | (-14.1, 2.4) |
|  | Pre-HSCT to 100-day post-HSCT | 3.9 | (-5.9, 13.7) | -6.4 | (-15.6, 3.2) |
|  | Baseline to 1-year post-HSCT | 2.4 | (-6.5, 11.3) | 0.9 | (-8.0, 9.8) |
|  | Pre-HSCT to 1-year post-HSCT | 2.4 | (-8.1, 12.9) | 0.5 | (-9.2, 10.2) |
|  | 100-day post-HSCT to 1-year post-HSCT | -1.5 | (-10.8, 7.8) | 6.7 | (-2.6, 16.1) |
| Triceps maximal voluntary contraction  (kg-force) | Baseline to Pre-HSCT | 1.1 | (-5.1, 7.2) | 1.7 | (-3.4, 6.8) |
|  | Baseline to 100-day post-HSCT | -3.1 | (-9.7, 3.4) | -4.8 | (-9.9, 0.3) |
|  | Pre-HSCT to 100-day post-HSCT | -4.2 | (-11.7, 3.3) | -6.5 | (-12.3, -0.8) |
|  | Baseline to 1-year post-HSCT | -1.3 | (-7.3, 4.7) | 1.1 | (-4.4, 6.6) |
|  | Pre-HSCT to 1-year post-HSCT | -2.3 | (-9.5, 4.9) | -0.6 | (-6.5, 5.3) |
|  | 100-day post-HSCT to 1-year post-HSCT | 1.9 | (-4.5, 8.2) | 5.9 | (0.2, 11.6) |
| Body fat  (%) | Baseline to Pre-HSCT | 1.4 | (-3.0, 5.7) | 0.4 | (-3.6, 4.4) |
|  | Baseline to 100-day post-HSCT | -3.2 | (-7.3, 0.8) | -5.7 | (-9.4, -1.9) |
|  | Pre-HSCT to 100-day post-HSCT | -4.6 | (-9.5, 0.3) | -6.1 | (-10.5, -1.6) |
|  | Baseline to 1-year post-HSCT | 0.1 | (-4.3, 4.4) | -0.3 | (-4.5, 4.0) |
|  | Pre-HSCT to 1-year post-HSCT | -1.3 | (-6.5, 4.0) | -0.6 | (-5.3, 4.0) |
|  | 100-day post-HSCT to 1-year post-HSCT | 3.3 | (-1.1, 7.8) | 5.4 | (1.0, 9.8) |
| Waist circumference  (cm) | Baseline to Pre-HSCT | -0.6 | (-6.5, 5.3) | 0.4 | (-5.1, 5.9) |
|  | Baseline to 100-day post-HSCT | -6.0 | (-11.6, -0.4) | -4.8 | (-10.0, 0.4) |
|  | Pre-HSCT to 100-day post-HSCT | -5.4 | (-12.2, 1.3) | -5.2 | (-11.4, 0.9) |
|  | Baseline to 1-year post-HSCT | -4.2 | (-10.2, 1.8) | -0.8 | (-6.7, 5.1) |
|  | Pre-HSCT to 1-year post-HSCT | -3.6 | (-10.9, 3.6) | -1.2 | (-7.7, 5.3) |
|  | 100-day post-HSCT to 1-year post-HSCT | 1.8 | (-4.4, 8) | 4.1 | (-2.1, 10.2) |
| *estimates between time points are ratios. | | | | | |

| Table S5: Mean estimates ± SE and between-group differences for patient-reported outcomes at each time point. | | | | | |
| --- | --- | --- | --- | --- | --- |
| **Outcome** | **Time point** | **Group** | | **Between-group contrasts**  **(Intervention – Control)** | |
|  |  | **Intervention (n=15)**  Mean estimates ± SE | **Control (n=15)**  Mean estimates ± SE | **Δ** | **(95% CI)** |
| QLQ-C30 summary | Baseline | 79.2 ± 3.1 | 84.0 ± 3.1 | -4.8 | (-13.7, 4.0) |
|  | Pre-HSCT | 78.4 ± 4.5 | 83.5 ± 4.2 | -5.1 | (-17.3, 7.2) |
|  | 100-day post-HSCT | 78.7 ± 4.2 | 85.0 ± 4.0 | -6.3 | (-17.9, 5.3) |
|  | 1-year post-HSCT | 87.3 ± 4.5 | 86.7 ± 4.5 | 0.6 | (-12.0, 13.2) |
| QLQ-C30 global health status | Baseline | 67.2 ± 5.8 | 68.9 ± 5.8 | -1.7 | (-18.2, 14.9) |
|  | Pre-HSCT | 63.6 ± 7.1 | 65.9 ± 6.8 | -2.3 | (-22.0, 17.5) |
|  | 100-day post-HSCT | 67.1 ± 6.9 | 65.0 ± 6.6 | 2.1 | (-17.1, 21.2) |
|  | 1-year post-HSCT | 69.5 ± 7.1 | 71.2 ± 7.1 | -1.7 | (-21.8, 18.4) |
| QLQ-C30 physical functioning | Baseline | 84.0 ± 4.4 | 87.9 ± 4.4 | -3.9 | (-16.3, 8.5) |
|  | Pre-HSCT | 79.3 ± 6.7 | 90.0 ± 6.7 | -10.7 | (-29.6, 8.1) |
|  | 100-day post-HSCT | 69.9 ± 6.7 | 87.0 ± 6.3 | -17.1 | (-35.4, 1.2) |
|  | 1-year post-HSCT | 86.0 ± 7.3 | 87.1 ± 7.3 | -1.0 | (-21.6, 19.5) |
| QLQ-C30 role functioning | Baseline | 73.3 ± 6.4 | 80.0 ± 6.4 | -6.7 | (-24.8, 11.5) |
|  | Pre-HSCT | 61.6 ± 9.7 | 93.4 ± 9.7 | -31.8 | (-59.3, -4.4) |
|  | 100-day post-HSCT | 60.1 ± 9.8 | 80.8 ± 9.1 | -20.7 | (-47.3, 6.0) |
|  | 1-year post-HSCT | 78.7 ± 10.6 | 86.6 ± 10.6 | -8.0 | (-37.8, 21.9) |
| QLQ-C30 emotional functioning | Baseline | 76.1 ± 4.8 | 86.7 ± 4.8 | -10.6 | (-24.2, 3.1) |
|  | Pre-HSCT | 74.6 ± 6.7 | 78.4 ± 6.3 | -3.8 | (-22.1, 14.5) |
|  | 100-day post-HSCT | 84.0 ± 6.3 | 86.3 ± 6.0 | -2.4 | (-19.8, 15.1) |
|  | 1-year post-HSCT | 80.8 ± 6.7 | 78.7 ± 6.7 | 2.1 | (-16.8, 20.9) |
| QLQ-C30 cognitive functioning | Baseline | 78.9 ± 4.8 | 91.1 ± 4.8 | -12.2 | (-25.8, 1.3) |
|  | Pre-HSCT | 79.2 ± 7.7 | 80.1 ± 7.1 | -0.9 | (-21.8, 19.9) |
|  | 100-day post-HSCT | 86.0 ± 7.1 | 88.4 ± 6.6 | -2.5 | (-21.9, 17.0) |
|  | 1-year post-HSCT | 88.9 ± 7.7 | 79.7 ± 7.7 | 9.1 | (-12.5, 30.8) |
| QLQ-C30 social functioning | Baseline | 61.1 ± 7.4 | 68.9 ± 7.4 | -7.8 | (-28.9, 13.3) |
|  | Pre-HSCT | 57.9 ± 12.3 | 80.6 ± 11.3 | -22.7 | (-55.9, 10.5) |
|  | 100-day post-HSCT | 62.9 ± 11.3 | 70.4 ± 10.5 | -7.5 | (-38.4, 23.3) |
|  | 1-year post-HSCT | 92.3 ± 12.3 | 82.8 ± 12.2 | 9.5 | (-25.0, 44.0) |
| MSAS Global Distress Index | Baseline | 0.9 ± 0.2 | 0.7 ± 0.2 | 0.2 | (-0.3, 0.7) |
|  | Pre-HSCT | 0.8 ± 0.2 | 0.8 ± 0.2 | 0.0 | (-0.6, 0.5) |
|  | 100-day post-HSCT | 0.7 ± 0.2 | 0.7 ± 0.2 | 0.1 | (-0.5, 0.6) |
|  | 1-year post-HSCT | 0.6 ± 0.2 | 0.9 ± 0.2 | -0.3 | (-0.9, 0.3) |
| MSAS Physical | Baseline | 0.6 ± 0.1 | 0.5 ± 0.1 | 0.1 | (-0.3, 0.5) |
|  | Pre-HSCT | 0.4 ± 0.2 | 0.6 ± 0.2 | -0.2 | (-0.6, 0.3) |
|  | 100-day post-HSCT | 0.6 ± 0.2 | 0.5 ± 0.1 | 0.1 | (-0.3, 0.5) |
|  | 1-year post-HSCT | 0.4 ± 0.2 | 0.6 ± 0.2 | -0.2 | (-0.6, 0.3) |
| MSAS Psychological | Baseline | 1.0 ± 0.2 | 0.9 ± 0.2 | 0.1 | (-0.5, 0.7) |
|  | Pre-HSCT | 1.0 ± 0.3 | 1.0 ± 0.3 | 0.0 | (-0.8, 0.8) |
|  | 100-day post-HSCT | 0.4 ± 0.3 | 0.8 ± 0.3 | -0.4 | (-1.1, 0.4) |
|  | 1-year post-HSCT | 0.8 ± 0.3 | 1.0 ± 0.3 | -0.2 | (-1.0, 0.6) |
| FACT-F | Baseline | 33.5 ± 2.7 | 39.7 ± 2.7 | -6.2 | (-13.9, 1.5) |
|  | Pre-HSCT | 32.2 ± 3.8 | 40.8 ± 3.6 | -8.6 | (-19.0, 1.8) |
|  | 100-day post-HSCT | 36.4 ± 3.6 | 41.5 ± 3.4 | -5.0 | (-15.0, 4.9) |
|  | 1-year post-HSCT | 42.2 ± 3.8 | 41.1 ± 3.8 | 1.1 | (-9.6, 11.8) |
| MFI | Baseline | 61.7 ± 1.5 | 62.4 ± 1.5 | -0.7 | (-4.9, 3.4) |
|  | Pre-HSCT | 57.6 ± 2.3 | 63.3 ± 2.3 | -5.7 | (-12.0, 0.7) |
|  | 100-day post-HSCT | 63.3 ± 2.3 | 61.5 ± 2.1 | 1.8 | (-4.4, 8.0) |
|  | 1-year post-HSCT | 63.7 ± 2.5 | 61.0 ± 2.7 | 2.8 | (-4.5, 10.1) |
| MFI general fatigue | Baseline | 10.7 ± 0.5 | 11.0 ± 0.5 | -0.3 | (-1.8, 1.2) |
|  | Pre-HSCT | 10.6 ± 0.8 | 11.4 ± 0.8 | -0.7 | (-3.1, 1.6) |
|  | 100-day post-HSCT | 11.2 ± 0.8 | 10.6 ± 0.8 | 0.7 | (-1.6, 2.9) |
|  | 1-year post-HSCT | 11.7 ± 0.9 | 10.8 ± 0.9 | 0.8 | (-1.7, 3.4) |
| MFI physical fatigue | Baseline | 13.7 ± 0.5 | 13.0 ± 0.5 | 0.7 | (-0.7, 2.1) |
|  | Pre-HSCT | 13.2 ± 0.8 | 13.3 ± 0.8 | -0.1 | (-2.3, 2.0) |
|  | 100-day post-HSCT | 13.9 ± 0.8 | 13.4 ± 0.7 | 0.5 | (-1.6, 2.6) |
|  | 1-year post-HSCT | 12.7 ± 0.8 | 13 ± 0.8 | -0.3 | (-2.6, 2.0) |
| MFI reduced activity | Baseline | 11.4 ± 0.5 | 11.1 ± 0.5 | 0.3 | (-1.0, 1.6) |
|  | Pre-HSCT | 10.9 ± 0.7 | 12.1 ± 0.7 | -1.1 | (-3.2, 0.9) |
|  | 100-day post-HSCT | 11.7 ± 0.7 | 11.0 ± 0.7 | 0.8 | (-1.2, 2.7) |
|  | 1-year post-HSCT | 12.5 ± 0.8 | 11.6 ± 0.9 | 0.9 | (-1.4, 3.3) |
| MFI reduced motivation | Baseline | 13.4 ± 0.5 | 13.9 ± 0.5 | -0.5 | (-1.9, 0.8) |
|  | Pre-HSCT | 11.3 ± 0.8 | 14.2 ± 0.8 | -2.8 | (-5.0, -0.7) |
|  | 100-day post-HSCT | 14.3 ± 0.8 | 13.4 ± 0.7 | 0.9 | (-1.2, 3.0) |
|  | 1-year post-HSCT | 13.6 ± 0.8 | 13.2 ± 1 | 0.4 | (-2.2, 2.9) |
| MFI mental fatigue | Baseline | 12.5 ± 0.6 | 13.4 ± 0.6 | -0.9 | (-2.6, 0.8) |
|  | Pre-HSCT | 12.0 ± 0.9 | 12.8 ± 0.9 | -0.8 | (-3.5, 1.8) |
|  | 100-day post-HSCT | 12.4 ± 0.9 | 13.5 ± 0.9 | -1.1 | (-3.7, 1.4) |
|  | 1-year post-HSCT | 13.4 ± 1.0 | 13.3 ± 1.0 | 0.1 | (-2.8, 3.0) |
| PHQ | Baseline | 5.6 ± 1.0 | 3.1 ± 1.0 | 2.4 | (-0.3, 5.2) |
|  | Pre-HSCT | 5.0 ± 1.4 | 4.7 ± 1.4 | 0.3 | (-3.6, 4.3) |
|  | 100-day post-HSCT | 4.4 ± 1.5 | 2.4 ± 1.3 | 2.0 | (-2.0, 6.0) |
|  | 1-year post-HSCT | 3.4 ± 1.5 | 3.5 ± 1.5 | 0.0 | (-4.3, 4.2) |
| GAD7 | Baseline | 5.2 ± 1.1 | 3.0 ± 1.1 | 2.2 | (-1.0, 5.3) |
|  | Pre-HSCT | 6.0 ± 1.4 | 4.3 ± 1.4 | 1.7 | (-2.2, 5.6) |
|  | 100-day post-HSCT | 2.3 ± 1.4 | 2.8 ± 1.3 | -0.5 | (-4.3, 3.3) |
|  | 1-year post-HSCT | 2.9 ± 1.4 | 4.1 ± 1.4 | -1.2 | (-5.2, 2.9) |
| ESES | Baseline | 29.2 ± 1.5 | 33.7 ± 1.5 | -4.6 | (-8.9, -0.3) |
|  | Pre-HSCT | 26.9 ± 1.9 | 31.6 ± 1.9 | -4.7 | (-10.1, 0.7) |
|  | 100-day post-HSCT | 31.3 ± 1.9 | 33.1 ± 1.8 | -1.9 | (-7.2, 3.5) |
|  | 1-year post-HSCT | 28.2 ± 2.0 | 32.9 ± 2.0 | -4.6 | (-10.3, 1.1) |
| ESES: Exercise Self-Efficacy Scale; FACT-F: Functional assessment of Cancer Therapy-Fatigue; GAD7: Generalized Anxiety Disorder; MFI: Multidimentioanl Fatigue Inventory; MSAS: Memorial Symptom Assessment Scale; PHQ: Patient Health Questionnaire; QLQ-C30: European Organization for the Research and Treatmnet of Cancer Quality of Life Questionnaire. | | | | | |

| Table S6: Within-group differences across time points for patient-reported outcomes. | | | | | |
| --- | --- | --- | --- | --- | --- |
| **Outcome** | **Contrast** | **Group** | | | |
|  |  | **Intervention (n=15)** | | **Control (n=15)** | |
|  |  | **Estimate** | **95% CI** | **Estimate** | **95% CI** |
| QLQ-C30 summary | Baseline to Pre-HSCT | -0.8 | (-11.9, 10.3) | -0.5 | (-10.8, 9.7) |
|  | Baseline to 100-day post-HSCT | -0.4 | (-10.8, 9.9) | 1.0 | (-8.7, 10.7) |
|  | Pre-HSCT to 100-day post-HSCT | 0.3 | (-13, 13.7) | 1.6 | (-10.0, 13.1) |
|  | Baseline to 1-year post-HSCT | 8.1 | (-3.0, 19.3) | 2.7 | (-8.4, 13.7) |
|  | Pre-HSCT to 1-year post-HSCT | 8.9 | (-4.9, 22.7) | 3.2 | (-9.0, 15.5) |
|  | 100-day post-HSCT to 1-year post-HSCT | 8.6 | (-3.3, 20.4) | 1.7 | (-10, 13.3) |
| QLQ-C30 global health status | Baseline to Pre-HSCT | -3.6 | (-17.8, 10.6) | -3.0 | (-16.1, 10.1) |
|  | Baseline to 100-day post-HSCT | -0.2 | (-13.4, 13.1) | -3.9 | (-16.2, 8.5) |
|  | Pre-HSCT to 100-day post-HSCT | 3.4 | (-13.7, 20.5) | -0.9 | (-15.5, 13.8) |
|  | Baseline to 1-year post-HSCT | 2.3 | (-12.0, 16.5) | 2.3 | (-11.8, 16.3) |
|  | Pre-HSCT to 1-year post-HSCT | 5.9 | (-11.8, 23.5) | 5.3 | (-10.2, 20.7) |
|  | 100-day post-HSCT to 1-year post-HSCT | 2.4 | (-12.4, 17.3) | 6.1 | (-8.5, 20.8) |
| QLQ-C30 physical functioning | Baseline to Pre-HSCT | -4.7 | (-23.6, 14.2) | 2.1 | (-16.8, 21.0) |
|  | Baseline to 100-day post-HSCT | -14.1 | (-33.1, 4.8) | -0.9 | (-18.7, 16.9) |
|  | Pre-HSCT to 100-day post-HSCT | -9.4 | (-32.2, 13.4) | -3.0 | (-24.6, 18.6) |
|  | Baseline to 1-year post-HSCT | 2.0 | (-18.4, 22.5) | -0.8 | (-21.2, 19.6) |
|  | Pre-HSCT to 1-year post-HSCT | 6.8 | (-17.5, 31.1) | -2.9 | (-26.0, 20.2) |
|  | 100-day post-HSCT to 1-year post-HSCT | 16.2 | (-6.3, 38.7) | 0.1 | (-21.9, 22.1) |
| QLQ-C30 role functioning | Baseline to Pre-HSCT | -11.7 | (-38.9, 15.5) | 13.4 | (-13.8, 40.7) |
|  | Baseline to 100-day post-HSCT | -13.2 | (-40.5, 14.1) | 0.8 | (-24.9, 26.4) |
|  | Pre-HSCT to 100-day post-HSCT | -1.5 | (-34.3, 31.3) | -12.6 | (-43.7, 18.4) |
|  | Baseline to 1-year post-HSCT | 5.3 | (-24.1, 34.7) | 6.6 | (-22.7, 35.9) |
|  | Pre-HSCT to 1-year post-HSCT | 17.1 | (-17.9, 52) | -6.8 | (-40.0, 26.4) |
|  | 100-day post-HSCT to 1-year post-HSCT | 18.5 | (-13.7, 50.8) | 5.8 | (-25.7, 37.4) |
| QLQ-C30 emotional functioning | Baseline to Pre-HSCT | -1.5 | (-17.5, 14.5) | -8.2 | (-23.0, 6.5) |
|  | Baseline to 100-day post-HSCT | 7.8 | (-7.1, 22.8) | -0.4 | (-14.3, 13.5) |
|  | Pre-HSCT to 100-day post-HSCT | 9.3 | (-9.9, 28.6) | 7.9 | (-8.7, 24.5) |
|  | Baseline to 1-year post-HSCT | 4.6 | (-11.4, 20.6) | -8 | (-23.8, 7.9) |
|  | Pre-HSCT to 1-year post-HSCT | 6.1 | (-13.7, 26.0) | 0.3 | (-17.3, 17.8) |
|  | 100-day post-HSCT to 1-year post-HSCT | -3.2 | (-20.1, 13.7) | -7.6 | (-24.2, 9.0) |
| QLQ-C30 cognitive functioning | Baseline to Pre-HSCT | 0.3 | (-20.7, 21.2) | -11 | (-30.4, 8.3) |
|  | Baseline to 100-day post-HSCT | 7.1 | (-12.4, 26.5) | -2.7 | (-20.9, 15.5) |
|  | Pre-HSCT to 100-day post-HSCT | 6.8 | (-18.3, 31.9) | 8.3 | (-13.7, 30.4) |
|  | Baseline to 1-year post-HSCT | 10 | (-11.0, 30.9) | -11.4 | (-32.2, 9.4) |
|  | Pre-HSCT to 1-year post-HSCT | 9.7 | (-16.3, 35.7) | -0.4 | (-23.8, 23.1) |
|  | 100-day post-HSCT to 1-year post-HSCT | 2.9 | (-19.9, 25.7) | -8.7 | (-31.0, 13.6) |
| QLQ-C30 social functioning | Baseline to Pre-HSCT | -3.2 | (-37.1, 30.7) | 11.8 | (-19.6, 43.1) |
|  | Baseline to 100-day post-HSCT | 1.8 | (-29.7, 33.4) | 1.5 | (-28.0, 31.1) |
|  | Pre-HSCT to 100-day post-HSCT | 5.0 | (-35.6, 45.6) | -10.2 | (-46.0, 25.6) |
|  | Baseline to 1-year post-HSCT | 31.2 | (-2.7, 65.1) | 13.9 | (-19.9, 47.7) |
|  | Pre-HSCT to 1-year post-HSCT | 34.4 | (-7.7, 76.5) | 2.1 | (-36.1, 40.3) |
|  | 100-day post-HSCT to 1-year post-HSCT | 29.4 | (-7.8, 66.6) | 12.3 | (-24.0, 48.7) |
| MSAS Global Distress Index | Baseline to Pre-HSCT | -0.1 | (-0.4, 0.2) | 0.1 | (-0.2, 0.4) |
|  | Baseline to 100-day post-HSCT | -0.2 | (-0.5, 0.2) | -0.1 | (-0.4, 0.3) |
|  | Pre-HSCT to 100-day post-HSCT | -0.1 | (-0.5, 0.3) | -0.1 | (-0.5, 0.2) |
|  | Baseline to 1-year post-HSCT | -0.3 | (-0.7, 0.1) | 0.2 | (-0.2, 0.5) |
|  | Pre-HSCT to 1-year post-HSCT | -0.2 | (-0.6, 0.2) | 0.1 | (-0.3, 0.5) |
|  | 100-day post-HSCT to 1-year post-HSCT | -0.1 | (-0.5, 0.3) | 0.2 | (-0.2, 0.6) |
| MSAS Physical | Baseline to Pre-HSCT | -0.2 | (-0.5, 0.0) | 0.0 | (-0.3, 0.3) |
|  | Baseline to 100-day post-HSCT | 0.0 | (-0.3, 0.3) | 0.0 | (-0.3, 0.3) |
|  | Pre-HSCT to 100-day post-HSCT | 0.2 | (-0.1, 0.6) | 0.0 | (-0.4, 0.3) |
|  | Baseline to 1-year post-HSCT | -0.2 | (-0.5, 0.1) | 0.0 | (-0.3, 0.4) |
|  | Pre-HSCT to 1-year post-HSCT | 0.0 | (-0.3, 0.4) | 0.0 | (-0.3, 0.4) |
|  | 100-day post-HSCT to 1-year post-HSCT | -0.2 | (-0.5, 0.1) | 0.1 | (-0.3, 0.4) |
| MSAS Psychological | Baseline to Pre-HSCT | 0.0 | (-0.6, 0.7) | 0.1 | (-0.5, 0.7) |
|  | Baseline to 100-day post-HSCT | -0.6 | (-1.2, 0.0) | -0.1 | (-0.7, 0.4) |
|  | Pre-HSCT to 100-day post-HSCT | -0.7 | (-1.5, 0.1) | -0.3 | (-1.0, 0.4) |
|  | Baseline to 1-year post-HSCT | -0.3 | (-0.9, 0.4) | 0.1 | (-0.6, 0.7) |
|  | Pre-HSCT to 1-year post-HSCT | -0.3 | (-1.1, 0.5) | 0.0 | (-0.8, 0.7) |
|  | 100-day post-HSCT to 1-year post-HSCT | 0.4 | (-0.3, 1.1) | 0.2 | (-0.5, 0.9) |
| FACT-F | Baseline to Pre-HSCT | -1.3 | (-10.5, 7.9) | 1.1 | (-7.4, 9.5) |
|  | Baseline to 100-day post-HSCT | 2.9 | (-5.6, 11.5) | 1.8 | (-6.2, 9.7) |
|  | Pre-HSCT to 100-day post-HSCT | 4.2 | (-6.8, 15.3) | 0.7 | (-8.8, 10.2) |
|  | Baseline to 1-year post-HSCT | 8.7 | (-0.5, 17.9) | 1.4 | (-7.7, 10.5) |
|  | Pre-HSCT to 1-year post-HSCT | 10.0 | (-1.4, 21.4) | 0.3 | (-9.8, 10.4) |
|  | 100-day post-HSCT to 1-year post-HSCT | 5.8 | (-4.0, 15.5) | -0.4 | (-9.9, 9.1) |
| MFI | Baseline to Pre-HSCT | -4.0 | (-10.4, 2.4) | 0.9 | (-5.5, 7.3) |
|  | Baseline to 100-day post-HSCT | 1.6 | (-4.8, 8.0) | -0.9 | (-7.0, 5.1) |
|  | Pre-HSCT to 100-day post-HSCT | 5.6 | (-2.1, 13.3) | -1.8 | (-9.1, 5.5) |
|  | Baseline to 1-year post-HSCT | 2.1 | (-4.8, 9.0) | -1.4 | (-9.0, 6.1) |
|  | Pre-HSCT to 1-year post-HSCT | 6.1 | (-2.1, 14.3) | -2.3 | (-10.8, 6.1) |
|  | 100-day post-HSCT to 1-year post-HSCT | 0.5 | (-7.1, 8.1) | -0.5 | (-8.6, 7.6) |
| MFI general fatigue | Baseline to Pre-HSCT | 0.0 | (-2.5, 2.4) | 0.4 | (-2.1, 2.8) |
|  | Baseline to 100-day post-HSCT | 0.6 | (-1.9, 3.0) | -0.4 | (-2.7, 1.8) |
|  | Pre-HSCT to 100-day post-HSCT | 0.6 | (-2.3, 3.5) | -0.8 | (-3.6, 2.0) |
|  | Baseline to 1-year post-HSCT | 1.0 | (-1.6, 3.6) | -0.2 | (-2.8, 2.5) |
|  | Pre-HSCT to 1-year post-HSCT | 1.1 | (-2.1, 4.2) | -0.5 | (-3.5, 2.5) |
|  | 100-day post-HSCT to 1-year post-HSCT | 0.5 | (-2.5, 3.4) | 0.3 | (-2.6, 3.2) |
| MFI physical fatigue | Baseline to Pre-HSCT | -0.5 | (-2.6, 1.7) | 0.3 | (-1.8, 2.5) |
|  | Baseline to 100-day post-HSCT | 0.2 | (-1.9, 2.4) | 0.4 | (-1.6, 2.4) |
|  | Pre-HSCT to 100-day post-HSCT | 0.7 | (-1.9, 3.3) | 0.1 | (-2.4, 2.6) |
|  | Baseline to 1-year post-HSCT | -1.0 | (-3.3, 1.3) | 0.0 | (-2.4, 2.3) |
|  | Pre-HSCT to 1-year post-HSCT | -0.5 | (-3.3, 2.3) | -0.3 | (-3.0, 2.3) |
|  | 100-day post-HSCT to 1-year post-HSCT | -1.3 | (-3.8, 1.3) | -0.4 | (-3.0, 2.1) |
| MFI reduced activity | Baseline to Pre-HSCT | -0.5 | (-2.6, 1.6) | 1.0 | (-1.1, 3.1) |
|  | Baseline to 100-day post-HSCT | 0.3 | (-1.8, 2.4) | -0.1 | (-2.1, 1.9) |
|  | Pre-HSCT to 100-day post-HSCT | 0.8 | (-1.7, 3.3) | -1.1 | (-3.5, 1.3) |
|  | Baseline to 1-year post-HSCT | 1.1 | (-1.1, 3.4) | 0.5 | (-1.9, 3.0) |
|  | Pre-HSCT to 1-year post-HSCT | 1.6 | (-1.1, 4.3) | -0.5 | (-3.2, 2.3) |
|  | 100-day post-HSCT to 1-year post-HSCT | 0.8 | (-1.7, 3.3) | 0.6 | (-2.0, 3.3) |
| MFI reduced motivation | Baseline to Pre-HSCT | -2.1 | (-4.5, 0.3) | 0.2 | (-2.2, 2.6) |
|  | Baseline to 100-day post-HSCT | 0.9 | (-1.5, 3.3) | -0.5 | (-2.8, 1.8) |
|  | Pre-HSCT to 100-day post-HSCT | 3.0 | (0.1, 5.9) | -0.7 | (-3.5, 2.0) |
|  | Baseline to 1-year post-HSCT | 0.2 | (-2.4, 2.8) | -0.7 | (-3.5, 2.1) |
|  | Pre-HSCT to 1-year post-HSCT | 2.3 | (-0.8, 5.3) | -0.9 | (-4.1, 2.3) |
|  | 100-day post-HSCT to 1-year post-HSCT | -0.7 | (-3.7, 2.3) | -0.2 | (-3.3, 2.9) |
| MFI mental fatigue | Baseline to Pre-HSCT | -0.5 | (-3.3, 2.2) | -0.6 | (-3.3, 2.2) |
|  | Baseline to 100-day post-HSCT | -0.2 | (-2.9, 2.6) | 0.1 | (-2.5, 2.7) |
|  | Pre-HSCT to 100-day post-HSCT | 0.4 | (-3.0, 3.7) | 0.7 | (-2.5, 3.8) |
|  | Baseline to 1-year post-HSCT | 0.9 | (-2.1, 3.8) | -0.1 | (-3.1, 2.9) |
|  | Pre-HSCT to 1-year post-HSCT | 1.4 | (-2.1, 4.9) | 0.5 | (-2.9, 3.9) |
|  | 100-day post-HSCT to 1-year post-HSCT | 1.0 | (-2.3, 4.4) | -0.2 | (-3.4, 3.1) |
| PHQ | Baseline to Pre-HSCT | -0.6 | (-4.3, 3.1) | 1.5 | (-2.2, 5.2) |
|  | Baseline to 100-day post-HSCT | -1.1 | (-5.2, 2.9) | -0.7 | (-4.2, 2.8) |
|  | Pre-HSCT to 100-day post-HSCT | -0.6 | (-5.4, 4.2) | -2.2 | (-6.4, 1.9) |
|  | Baseline to 1-year post-HSCT | -2.1 | (-6.2, 1.9) | 0.4 | (-3.6, 4.3) |
|  | Pre-HSCT to 1-year post-HSCT | -1.6 | (-6.4, 3.2) | -1.2 | (-5.6, 3.3) |
|  | 100-day post-HSCT to 1-year post-HSCT | -1 | (-5.4, 3.4) | 1.1 | (-3.2, 5.3) |
| GAD7 | Baseline to Pre-HSCT | 0.8 | (-2.2, 3.8) | 1.2 | (-1.9, 4.4) |
|  | Baseline to 100-day post-HSCT | -2.9 | (-5.9, 0.1) | -0.2 | (-3.1, 2.6) |
|  | Pre-HSCT to 100-day post-HSCT | -3.7 | (-7.3, -0.1) | -1.5 | (-4.9, 2.0) |
|  | Baseline to 1-year post-HSCT | -2.3 | (-5.5, 1.0) | 1.1 | (-2.2, 4.3) |
|  | Pre-HSCT to 1-year post-HSCT | -3.1 | (-6.9, 0.8) | -0.2 | (-3.8, 3.4) |
|  | 100-day post-HSCT to 1-year post-HSCT | 0.6 | (-2.8, 4) | 1.3 | (-2.1, 4.7) |
| ESES | Baseline to Pre-HSCT | -2.3 | (-6.6, 2.1) | -2.2 | (-6.4, 2.0) |
|  | Baseline to 100-day post-HSCT | 2.1 | (-2.3, 6.5) | -0.6 | (-4.6, 3.4) |
|  | Pre-HSCT to 100-day post-HSCT | 4.4 | (-0.7, 9.5) | 1.6 | (-3.2, 6.3) |
|  | Baseline to 1-year post-HSCT | -0.9 | (-5.7, 3.8) | -0.9 | (-5.4, 3.7) |
|  | Pre-HSCT to 1-year post-HSCT | 1.4 | (-4.1, 6.8) | 1.3 | (-3.7, 6.3) |
|  | 100-day post-HSCT to 1-year post-HSCT | -3 | (-7.8, 1.8) | -0.3 | (-5.0, 4.5) |
| ESES: Exercise Self-Efficacy Scale; FACT-F: Functional assessment of Cancer Therapy-Fatigue; GAD7: Generalized Anxiety Disorder; MFI: Multidimentioanl Fatigue Inventory; MSAS: Memorial Symptom Assessment Scale; PHQ: Patient Health Questionnaire; QLQ-C30: European Organization for the Research and Treatmnet of Cancer Quality of Life Questionnaire. | | | | | |
